# SARS-CoV-2 ORF3a induces COVID-19-associated kidney injury through HMGB1-mediated cytokine production

**DOI:** 10.1128/mbio.02308-24

**Published:** 2024-09-30

**Authors:** Chenyu Zhang, Volodymyr Gerzanich, Ruth Cruz-Cosme, Jiantao Zhang, Orest Tsymbalyuk, Cigdem Tosun, Bhargava Teja Sallapalli, Dongxiao Liu, Kaspar Keledjian, John C. Papadimitriou, Cinthia B. Drachenberg, Mohamed Nasr, Yanjin Zhang, Qiyi Tang, J. Marc Simard, Richard Y. Zhao

**Affiliations:** 1 Department of Pathology, University of Maryland School of Medicine, Baltimore, Maryland, USA; 2 Department of Neurosurgery, University of Maryland School of Medicine, Baltimore, Maryland, USA; 3 Department of Microbiology, Howard University College of Medicine, Washington, DC, USA; 4 Department of Veterinary Medicine, University of Maryland, College Park, Maryland, USA; 5 Division of AIDS, NIAID, NIH, Drug Development and Clinical Sciences Branch, Bethesda, Maryland, USA; 6 Research and Development Service, VA Maryland Health Care System, Baltimore, Maryland, USA; 7 Department of Microbiology and Immunology, University of Maryland School of Medicine, Baltimore, Maryland, USA; 8 Institute of Human Virology, University of Maryland School of Medicine, Baltimore, Maryland, USA; 9 Institute of Global Health, University of Maryland School of Medicine, Baltimore, Maryland, USA; Johns Hopkins Bloomberg School of Public Health, Baltimore, Maryland, USA

**Keywords:** SARS-CoV-2, ORF3a, CAKI, HMGB1, glycyrrhizin, NF-kB, TNFα and IL-6, viral infection, K18-hACE2 mice

## Abstract

The primary challenge posed by severe acute respiratory syndrome coronavirus 2 (SARS-CoV-2) infection is COVID-19-related mortality, often exacerbated by additional medical complications, such as COVID-19-associated kidney injuries (CAKIs). Up to half of COVID-19 patients experience kidney complications, with those facing acute respiratory failure and kidney injury having the worst overall prognosis. Despite the significant impact of CAKI on COVID-19-related mortality and its enduring effects in long COVID, the underlying causes and molecular mechanisms of CAKI remain elusive. In this study, we identified a functional relationship between the expression of the SARS-CoV-2 ORF3a protein and inflammation-driven apoptotic death of renal tubular epithelial cells in patients with CAKI. We demonstrate *in vitro* that ORF3a independently induces renal cell-specific apoptotic cell death, as evidenced by the elevation of kidney injury molecule-1 (KIM-1) and the activation of NF-kB-mediated proinflammatory cytokine (TNFα and IL-6) production. By examining kidney tissues of SARS-CoV-2-infected K18-ACE2 transgenic mice, we observed a similar correlation between ORF3a-induced cytopathic changes and kidney injury. This correlation was further validated through reconstitution of the ORF3a effects via direct adenoviral injection into mouse kidneys. Through medicinal analysis, we identified a natural compound, glycyrrhizin (GL4419), which not only blocks viral replication in renal cells, but also mitigates ORF3a-induced renal cell death by inhibiting activation of a high mobility group box 1 (HMGB1) protein, leading to a reduction of KIM-1. Moreover, ORF3a interacts with HMGB1. Overproduction or downregulation of *hmgb1* expression results in correlative changes in renal cellular KIM-1 response and respective cytokine production, implicating a crucial role of HMGB1 in ORF3a-inflicted kidney injuries. Our data suggest a direct functional link between ORF3a and kidney injury, highlighting ORF3a as a unique therapeutic target contributing to CAKI.

**IMPORTANCE:**

The major challenge of severe acute respiratory syndrome coronavirus 2 (SARS-CoV-2) infection during the pandemic is COVID-19-related mortality, which has tragically claimed millions of lives. COVID-19-associated morbidity and mortality are often exacerbated by pre-existing medical conditions, such as chronic kidney diseases (CKDs), or the development of acute kidney injury (AKI) due to COVID-19, collectively known as COVID-19-associated kidney injuries (CAKIs). Patients who experience acute respiratory failure with CAKI have the poorest clinical outcomes, including increased mortality. Despite these alarming clinical findings, there is a critical gap in our understanding of the underlying causes of CAKI. Our study establishes a direct correlation between the expression of the SARS-CoV-2 viral ORF3a protein and kidney injury induced by ORF3a linking to CAKI. This functional relationship was initially observed in our clinical studies of COVID-19 patients with AKI and was further validated through animal and *in vitro* cellular studies, either by expressing ORF3a alone or in the context of viral infection. By elucidating this functional relationship and its underlying mechanistic pathways, our research deepens the understanding of COVID-19-associated kidney diseases and presents potential therapeutic avenues to address the healthcare challenges faced by individuals with underlying conditions.

## INTRODUCTION

Severe acute respiratory syndrome coronavirus 2 (SARS-CoV-2) is responsible for the COVID-19 pandemic, which has caused a staggering toll, with 1.2 million deaths in the United States and over 7 million worldwide. The primary challenge posed by SARS-CoV-2 infection is COVID-19-related mortality. Acute respiratory distress syndrome (ARDS) significantly contributes to this mortality, but COVID-19-associated morbidity and mortality are often worsened by additional medical complications, such as COVID-19-associated kidney injuries (CAKIs). CAKI tends to affect individuals with pre-existing chronic kidney diseases (CKDs) or those who develop acute kidney injury (AKI) following SARS-CoV-2 infection (1–4). Between 25 and 50% of COVID-19 patients experience kidney complications (5–7), with those facing both acute respiratory failure and kidney injury suffering the most severe outcomes, including death (8, 9). A comparable percentage of kidney complications is found among critically ill COVID-19 patients (10), with nearly half of those with AKI not fully recovered by discharge (7). Continued renal function decline in post-COVID patients suggests a long-COVID effect (11). Despite the significant impact of CAKI on COVID-19-related mortality (7) and its lasting effects in long COVID (3, 12), the underlying causes and molecular mechanisms of CAKI remain poorly understood.

Besides the lungs, SARS-CoV-2 also infects the kidneys via ACE2 and other receptors, such as CD209L/L-SIGN, CD209/DC-SIGN (13), and BSG/CD147 (14) in renal proximal tubular epithelial cells (RPTEC), which are vital for kidney structural integrity and function (15, 16). Both SARS-CoV-2 viral RNA and proteins have been detected in infected kidneys (10, 17), and viral particles recovered from kidneys have infected nonhuman primate RPTEC (18). Histopathological examinations of biopsy or postmortem human kidney tissues, including ours (19), show that injury in RPTEC is common in patients with CAKI (5, 19–21). These kidney-specific injuries can be verified by the activation of kidney injury molecule-1 (KIM-1), a well-established biomarker that is encoded by the *HAVCR1* gene for early detection of renal damage, which is minimally expressed in normal kidneys but highly expressed in injured RPTECs (22, 23). Renal cell injury is also associated with cellular oxidative stress, cell membrane damage, and inflammation-mediated cytokine release, such as TNFα and IL-6 (5, 19–21), leading to apoptosis and necrosis (24–26). However, the specific viral determinants responsible for CAKI remain undefined.

Through a genome-wide functional analysis of SARS-CoV-2 viral proteins, we identified a viral protein known as open-reading frame 3a (ORF3a) that induces oxidative stress and inflammation-mediated cell death in renal epithelial cells (27). Production of ORF3a protein in these cells not only induces oxidative stress but also triggers proinflammatory NF-kB-mediated cytokine production, including TNFα and IL-6 (27, 28), which are two strong and independent predictors of mortality in COVID-19 patients (29, 30). Other studies have reported similar findings (31, 32). This cytopathic effect seems to be a general property of ORF3a, present in both the original wild type (WT) and the subsequent naturally emerging mutant ORF3a proteins, although the levels of cytopathicity vary (28, 33). Note that ORF3a was selected not only because it induces oxidative stress and inflammation-mediated apoptotic cell death in renal epithelial cells, which are phenotypes observed in the kidney tissues of COVID-19 patients (19), but also because it meets all the criteria we established for identifying potential antiviral targets against SARS-CoV-2 infection (27). Specifically, ORF3a plays a pivotal role in viral pathogenesis, induces cytokine storms, damages cells and tissues, and contributes to the severity and mortality associated with COVID-19. For a comprehensive review of why targeting ORF3a has high therapeutic potential, see reference 34. It is well-established that cytokine storm, a severe immune overreaction, often leads to mortality that is associated with ARDS (35–37). Oxidative stress and hyper-inflammation also contribute to the severity of COVID-19 by causing cell and tissue damage (38, 39). ORF3a has been linked to the activation of the NLRP3 inflammasome (40–42), an inducer of cytokine storm (40, 41, 43, 44). In mouse models, SARS-CoV-2 infection caused dose-dependent and severe kidney damage, including tubular damage and focal tubular collapse, mimicking those observed in COVID-19 patients (26, 45). ORF3a deletion reduces the risk of cytokine storm and significantly reduces tissue pathology (46–51), suggesting the involvement of ORF3a involvement in tissue damage (51, 52). These observations imply that ORF3a might contribute to renal cell and tissue damage, as well as CAKI. Therefore, this study aimed to determine whether ORF3a contributes to CAKI and plays a specific role in causing kidney-specific cellular and tissue injuries.

In this study, we uncovered a functional relationship between the expression of the viral ORF3a protein and inflammation-driven apoptotic death of renal tubular epithelial cells in patients with CAKI. We validated this relationship by testing the cytopathic effect of ORF3a expression alone or in the context of viral infection in renal cells. To investigate whether a similar correlation between ORF3a and kidney injury exists in SARS-CoV-2-infected mice, we measured the presence of ORF3a protein and respective inflammation-mediated kidney injury in the kidney tissues of viral-infected K18-hACE transgenic mice. Finally, we determined the effect of ORF3a on kidney injury by directly injecting ORF3a-expressing adenovirus into mouse kidneys.

In searching for ORF3a inhibitors, through medicinal analysis, we identified a natural compound, glycyrrhizin (GL4419), a well-known inhibitor of high mobility group box 1 (HMGB1) (53). HMGB1 is a nuclear protein with multiple functions (54). Upon cellular stress or tissue damage, it acts as a damage-associated molecular pattern (DAMP) molecule, released from the nucleus to the cytoplasm and further secreted from damaged cells. Extracellular HMGB1 typically acts as a proinflammatory cytokine, regulating cellular immune responses (54). HMGB1 plays a crucial pathological role in various kidney diseases by activating cell membrane receptors and inducing inflammation (55). Therapeutic inhibition of HMGB1 by glycyrrhizin improves the long-term recovery of patients with AKI (56). Here, we show that glycyrrhizin blocks viral replication in renal cells and mitigates ORF3a-induced renal cell death, partly through HMGB1. This study presents a functional link between SARS-CoV-2 infection, ORF3a expression, and kidney injury, highlighting ORF3a as a possible therapeutic target for CAKI. Therefore, glycyrrhizin could potentially be used as a therapeutic drug to alleviate CAKI in SARS-CoV-2 infection.

## RESULTS

### Correlation of ORF3a expression with inflammation-associated renal cell apoptosis in COVID-19 patients with CAKI

**Fig 1:**
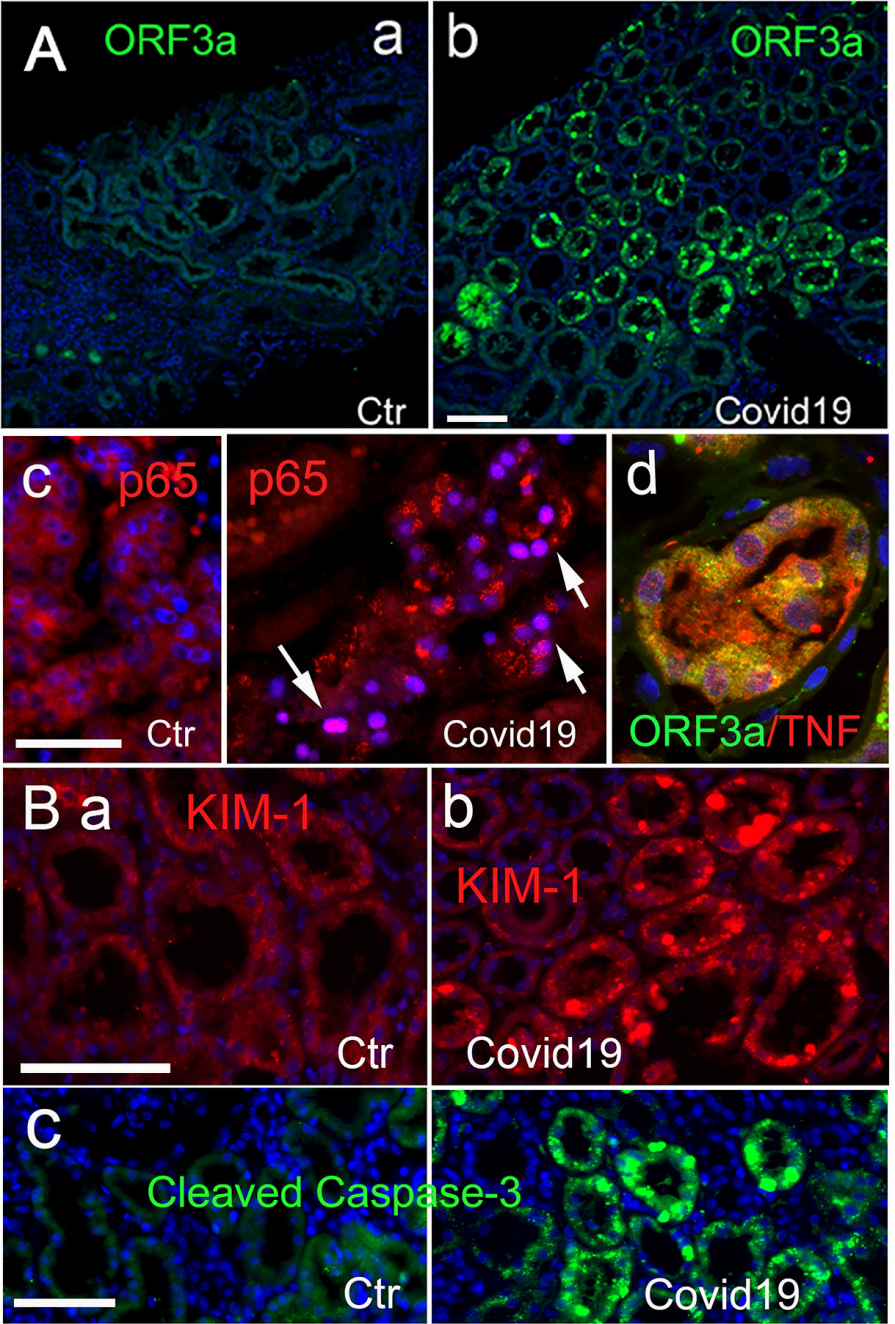
The correlation between SARS-CoV-2 infection and ORF3a expression that contributes to inflammation-associated apoptosis observed in RPTECs of the kidney in COVID-19 patients with CAKI. (**A)** Immunostaining of ORF3a in COVID-19-negative controls (Ctr, a) and COVID-19-positive subjects (b) (19). Comparative staining of NF-kB (p65) with DAPI between control and COVID-19-positive tissues (c). Arrows show nuclear **p65**, an indication of NF-kB activation. Co-immunolabeling for ORF3a and TNFα within RPTEC (d). **(B)** Comparative staining of KIM-1 (**a **vs b) and cleaved caspase 3 between control and COVID-19-positive tissues. The kidney shown is a representative of two COVID-19-positive and two control cases. Scale bars, 100 µM.

Our initial histopathologic examinations, utilizing fluorescent and electron microscopy on kidney biopsies from individuals with CAKI, indicated that RPTEC damage is strongly associated with oxidative stress and hyperinflammation-related cellular and tissue damage (19). Using the same biopsy samples, we investigated the potential relationship between ORF3a expression and CAKI using immunohistochemistry (IHC). Only non-specific background staining was detected in the control (Ctr) tissues with no specific labeling for ORF3a (Fig. 1A-a). In contrast, abundant ORF3a protein was detected predominantly in the RPTEC (Fig. 1A-b), which coincided with increased nuclear presence of the RelA/p65 subunit of NF-kB, an indicative of NF-kB activation (Fig. 1A-c, indicated by arrows), and with elevated TNFα (Fig. 1A-d). To determine if the observed tissue damage causes kidney-specific injury, we measured the protein level of KIM-1, a well-established kidney injury marker (22). A marked increase in KIM-1 was observed in COVID-19-positive tissues compared with controls (Fig. 1B-b vs. Fig. 1B-a). Furthermore, apoptotic cells, indicated by the presence of cleaved caspase 3 (cCasp3), were also observed (Fig. 1B-c). These findings demonstrate a positive association between ORF3a expression, NF-kB activation, and increased levels of TNFα in RPTEC (Fig. 1A and B), consistent with a kidney-specific injury response during SARS-CoV-2 infection (57).

### ORF3a independently induces renal cell-specific injuries and apoptotic cell death through NF-kB-mediated cytokine production

**Fig 2:**
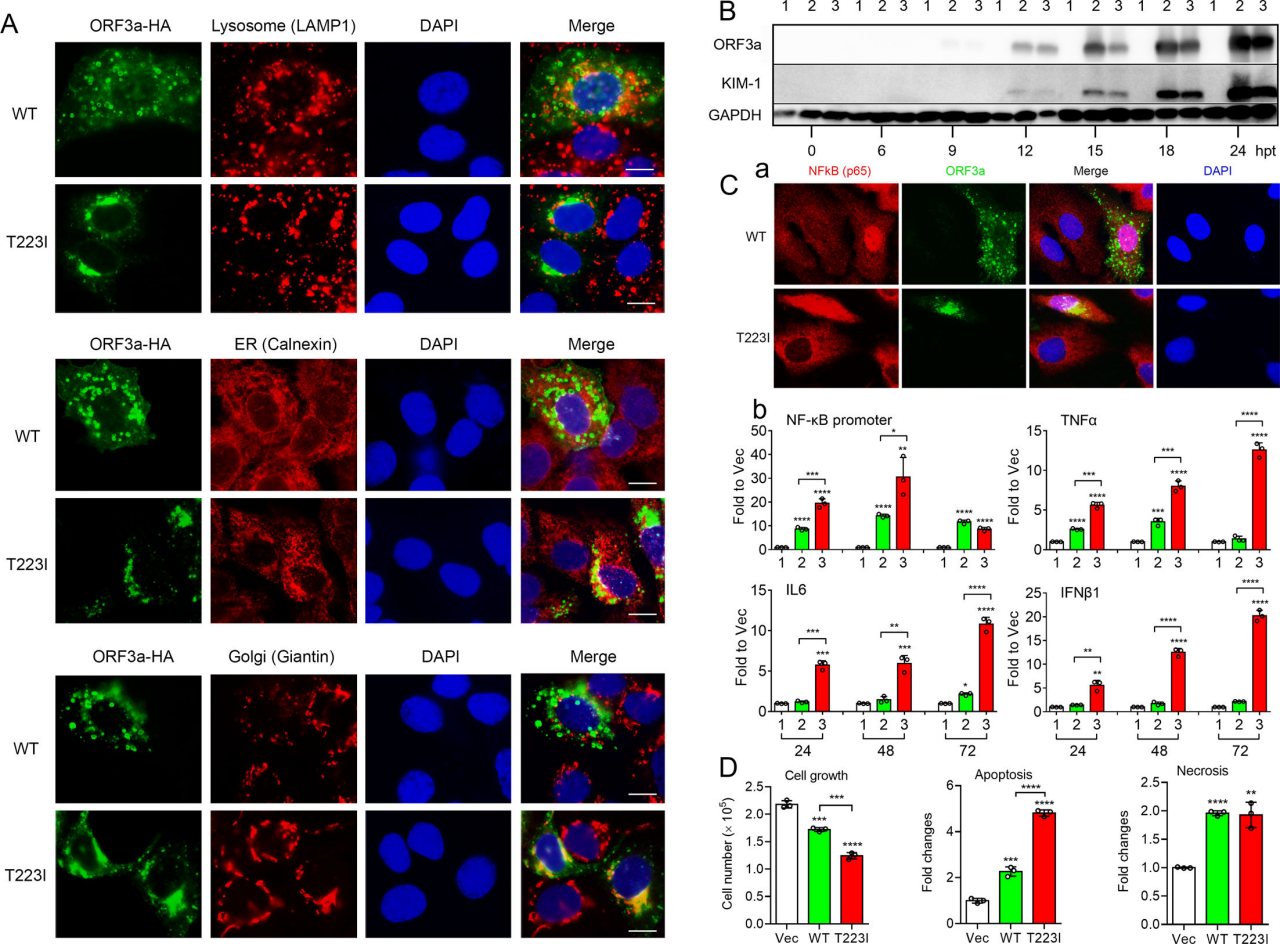
ORF3a independently induces renal cell-specific injuries and cell death through activation of NF-kB-mediated cytokine production. (**A)** Distinctive subcellular localization of WT and Omicron T223I mutant ORF3a proteins in HK2 cells. The WT protein is predominantly localized on the lysosomes as indicated by anti-LAMP-1 antibody and as we reported previously (28, 33). The T223I mutant mostly localizes in the ER and Golgi complex that are indicated by anti-calnexin and anti-giantin antibodies, respectively. (**B)** Time-course expression of WT and Omicron ORF3a T223I mutant shows concurrent elevation of KIM-1 protein levels in renal epithelial 293T cells (**A**). 293T cells were transfected with a WT or T223I *ORF3a*-carrying pCAG plasmid. Transfected cells were collected over time and as indicated in hours of post-transfection (hpt). Numeric numbers 1, 2, and 3 on the top of the Western blot represent vector-only control, WT, and T223I mutant ORF3a, respectively. (**C)** Expression of WT and T223I mutant proteins triggers NF-kB-mediated cytokine production. (**a)** Immunostaining of a RelA/p65 subunit of NF-kB in ORF3a-expression HK2 cells shows that nuclear entry of p65 is specifically associated with ORF3a, an indication of ORF3a-mediated NF-kB activation. Cells were examined at 24 hpt. (**b)** ORF3a induces NF-kB activation as measured by an *NF-kB* promoter firefly *luc* assay (27), and upregulation of cytokines TNFα, IL-6, and IFNβ1 by RT-qPCR. (**D)** Both WT and T223I ORF3a induce apoptosis and necrosis as measured by a RealTime-Glo apoptosis and necrosis assay (Promega). The levels of all markers were measured by RT-qPCR. Statistical significance: *, *P*  <  0.05; **, *P* <  0.01; ***, *P* <  0.001.

To ascertain if ORF3a alone could induce similar renal cell-specific injury and elevations in cellular proinflammatory immune responses as observed in human kidney tissues, we tested the effect of ORF3a protein production on human renal cell lines. Because our previous mutagenesis studies identified two distinctive types of ORF3a proteins based on their subcellular locations (28, 33), we wanted to examine both types of ORF3a proteins. We selected the WT and an Omicron-associated T223I mutant variant for testing (50). Given that SARS-CoV-2 primarily infects RPTEC (Fig. 1) (15, 16), we further examined the localization of these two ORF3a proteins in a human RPTEC HK2 cell line. As reported in our previous studies on 293T cells (28, 33) and shown in Fig. 2A, the WT ORF3a predominantly localizes to lysosomes, as detected by an anti-LAMP-1 antibody, with minimal presence in the endoplasmic reticulum (ER) (detected by an anti-calnexin antibody) or the Golgi apparatus (detected by an anti-Giantin antibody) in HK2 cells (Fig. 2A, top). In contrast, the T223I mutant predominantly localizes to the ER and Golgi apparatus, with minimal presence in lysosomes (Fig. 2A, bottom). This observation suggested that the WT and T223I mutant variant can be used as representative models for the two types of naturally occurring ORF3a proteins (28, 33).

Because KIM-1 is a well-established kidney injury marker (22), we first conducted a time-course experiment to examine how KIM-1 responds to ORF3a protein production. 293T cells were transfected with pCAG plasmids carrying either WT or T223I *ORF3a*. The transfected cells were collected at various time points as indicated. Initially, neither ORF3a proteins nor KIM-1 proteins were detectable in the early hours of post-transfection (hpt). However, ORF3a proteins became detectable at 9 hpt, followed by the appearance of KIM-1 proteins 3 h later, at 12 hpt (Fig. 2B).

Our earlier data suggested that ORF3a promotes NF-kB activation through transcriptional upregulation of RelA/p65 (27). Here, we further tested whether ORF3a facilitates the nuclear entry of RelA/p65, a hallmark of NF-kB activation (58). As shown in Fig. 2C-a, p65 proteins were dispersed in the cytoplasm in cells without ORF3a. However, a strong nuclear presence of the p65 proteins was seen in cells producing either WT or T223I mutant ORF3a. Consistent with our earlier findings (27, 28), the expression of ORF3a resulted in a significant increase in NF-kB promoter activity, peaking at 48 hpt (Fig. 2C-b, top left). This was followed by a gradual increase in the proinflammatory cytokines TNFα, IL-6, and IFNβ1 over a period of 72 hpt (Fig. 2C-b), ultimately resulting in apoptosis and necrosis (Fig. 2D). Notably, the T223I mutant protein had a significantly stronger effect on NF-kB-mediated cytokine productions than the WT protein.

### Correlation of ORF3a with kidney injury in SARS-CoV-2-infected K18-hACE2 transgenic mice

**Fig 3:**
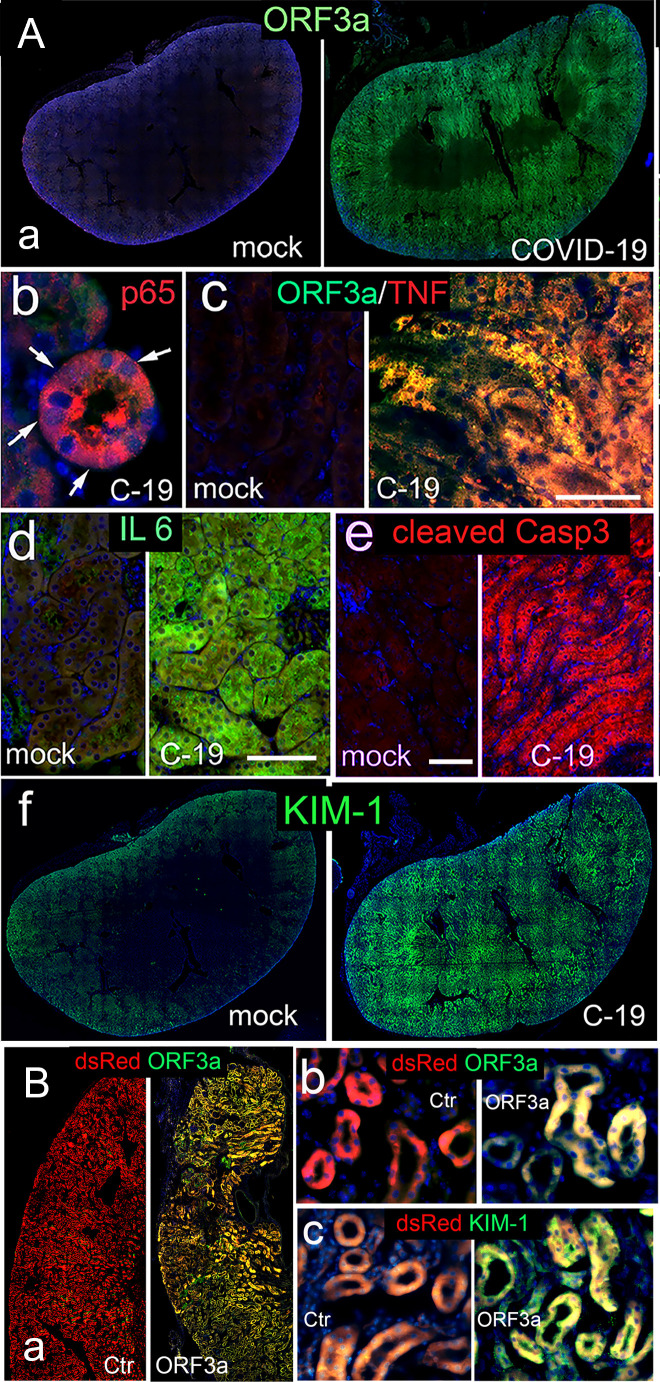
Correlation of ORF3a in kidney tissues of SARS-CoV-2-infected K18-ACE2 transgenic mice with ORF3a-induced cytopathicity and kidney injury, and reconstitution of the ORF3a effect by viral injection of Ad5-ORF3a. (**A)** SARS-CoV-2 infection in K18-hACE2 tg mice shows a correlation of ORF3a with inflammation-associated renal apoptosis in kidney. Immunostaining of ORF3a in a kidney cross-section of mock control (a, left) and a COVID-19 (C-19) mouse (a, right). Arrows point on p65 in “pink“ nuclei (b) indicating nuclear entry of the “red“ p65, indicative of NF-kB activation. Co-immunolabeling of ORF3a with TNFα (c), elevated IL-6 (d), cleaved Casp3 (e), and KIM-1 (f) between mock and C-19. (**B)** Abundant ORF3a protein production is observed in the left kidney of C57BL/6 mice injected with Ad5-DsRed-ORF3a (Ad5-ORF3a) compared with the Ad5-DsRed control (a). The ORF3a protein is predominantly localized in renal tubule cells (**b**), coinciding with elevated KIM-1 protein, a kidney-specific injury marker (**c**). Note that both Ad5 constructs carry a DsRed expression cassette in the E3 region of the adenoviral backbone. The expression cassette of ORF3a is driven by a CMV promoter located in the E1 region of the adenoviral genome. Scale bars, 100 µm.

To investigate whether the correlation between viral infection, ORF3a expression, and kidney injury observed in COVID-19 patients also exists in SARS-CoV-2-infected mice, we used a K18-hACE2 transgenic (Tg) mouse model (51, 59). Kidney tissues from SARS-CoV-2-infected K18-hACE2 Tg mice were collected on day 8 post-infection (p.i.). Compared with uninfected controls, abundant and widespread expression of ORF3a was observed in cross-sections of the entire kidney (Fig. 3A-a). Consistent with observations in human kidneys, SARS-CoV-2-infected cells in the RPTECs of these mice exhibited activated NF-kB (p65) (Fig. 3A-b, indicated by arrows), co-localization of ORF3a with TNFα (Fig. 3A-c), elevated IL-6 (Fig. 3A-d), expression of the cCasp3 (Fig. 3A-e), and increased KIM-1 levels (Fig. 3A-f) compared with controls.

To further investigate whether ORF3a alone can lead to kidney injury in mice, we delivered ORF3a into C57BL/6 mouse kidneys via direct injection of an adenoviral vector carrying ORF3a (Ad5-ORF3a). Adenovirus type 5 (Ad5) was used because of its ability to infect a broad group of different cell types. Specifically, Ad5-ORF3a was administered into the left kidney of mice using a well-established protocol (Fig. 3B) (32). This direct kidney injection method was chosen based on an early study demonstrating that ORF3a delivery via this route induced renal tubule injury in a mouse model (32). Control mice received Ad5-DsRed injected into the same kidney region. The mice were euthanized at 3 weeks p.i*.*, and the kidney tissues were collected and assessed for the levels of kidney injury compared with the controls. Our results showed abundant ORF3a protein in the injected kidneys (Fig. 3B-a, right) of RPTECs compared with the Ad5-DsRed control (Fig. 3B-a, left). Closer examination of RPTECs revealed a strong presence of ORF3a proteins in RPTECs (Fig. 3B-b) along with elevated level of the kidney-specific injury marker KIM-1 (Fig. 3B-c).

### Glycyrrhizin blocks viral replication and mitigates ORF3a-induced renal cell damage through HMGB1

**Fig 4:**
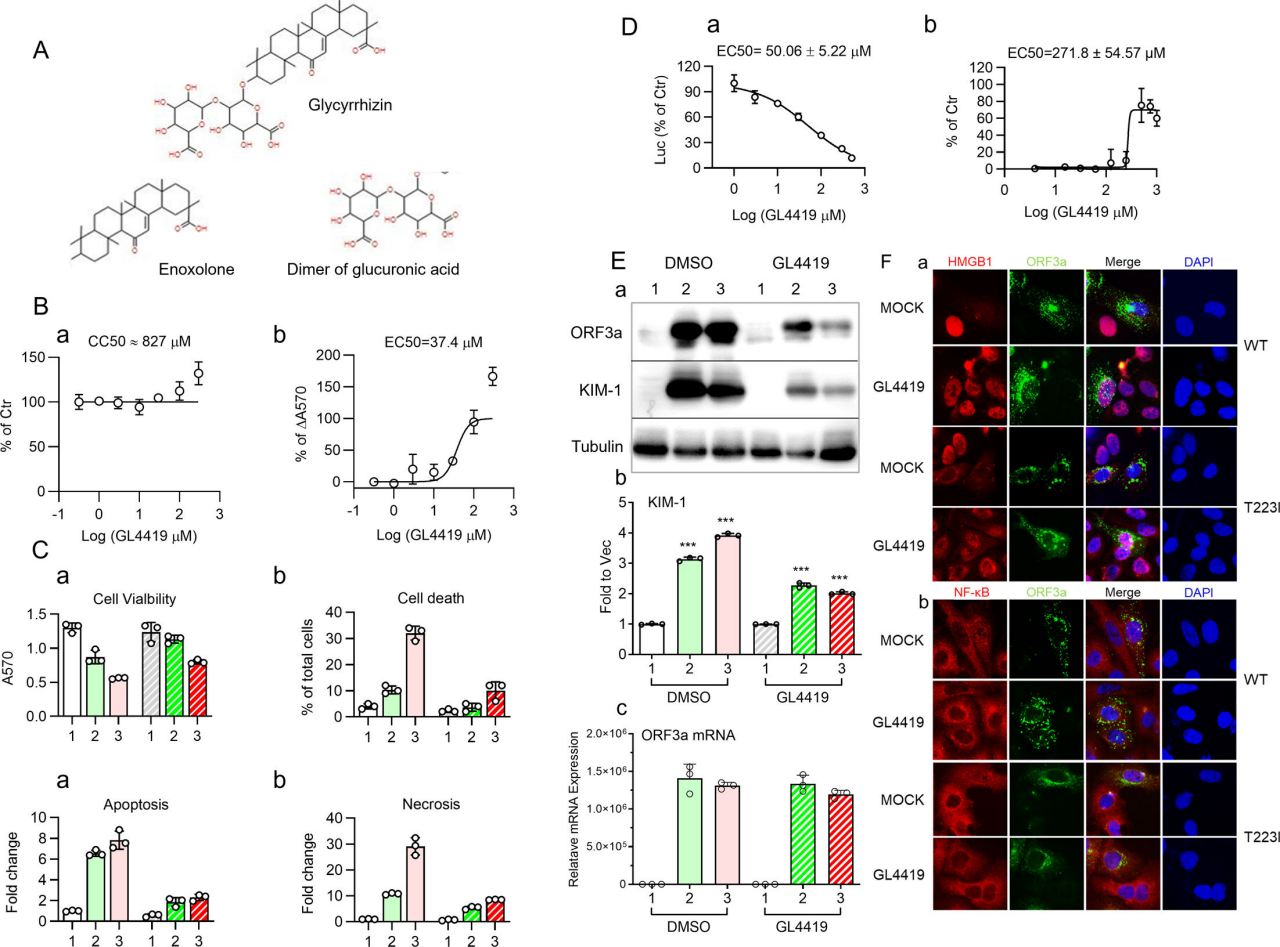
Glycyrrhizin (GL4419) blocks viral replication and mitigates ORF3a-induced renal cell damage through HMGB1. (**A)** The two chemical structures shown below glycyrrhizin are examples of glycyrrhizin derivatives we tested, including enoxolone (NSC-35347) and a dimer of glucuronic acid (NSC-2559). A list of glycyrrhizin-related compounds is listed in Table S1**.** (B) Treatment of GL4419 suppresses T223I ORF3a-induced cell death, displaying minimal cytotoxicity with a selectivity index of 22.11. (**C)** ORF3a mitigates ORF3a-induced apoptosis and necrosis. Tests were carried out in 293T cells at 24 hpt. Cell viability was measured by MTT, cell death by trypan blue, and apoptosis and necrosis by a RealTime-Glo Apoptosis and necrosis assay (Promega). The selectivity index was calculated by the ratio of EC_50_ over CC_50_ based on the MTT assay. (D-a). GL4419 inhibits viral replication, with an IC_50_ of 50.05 ± 5.22 µM, assessed in a stable BHK21 cell-based SARS-CoV-2 NanoLuc replicon system using a Nano-Glo Luciferase Assay (Promega). (D-b)**.** GL4419 inhibits SARS-CoV-2 replication in VeroE6 cells, exhibiting an EC_50_ of 271.8 ± 54.57. The WT USA-WA1/2020 virus was used for infection with titer of 100 TCID_50_, and the cells were collected at 48 hpi. (**E)** Treatment of ORF3a-expressing 293T cells with GL4419 (300 µM) reduces KIM-1 protein production possibly through protein degradation of ORF3a. 293T cells transfected with WT and T223I ORF3a were collected at 24 hpt. GL4419 dissolved in DMSO in the concentration of 300 µM was used. DMSO only was used as a negative control. western blotting results are shown in panel **a** with quantification shown in panel **b**. Results of RT-qPCR of transfected cells were collected at 48 hpt (**c**). **(F)** treatment of WT and T223I ORF3a-expressing HK2 cells with GL4419 block activation of NF-kB (indicated by the RelA/p65 subunit of NF-kB) and HMGB1. Expression of ORF3a without GL4419 treatment (mock with DMSO added) activates HMGB1 (**a**) and NF-kB (b) by promoting contrasting nuclear entry and nuclear exit, respectively. In contrast, adding GL4419 (300 µM) results in the retainment of HMGB1 in the nucleus (**a**) and NF-kB in the cytoplasm (**b**).

Glycyrrhizin (GL) is a well-documented natural compound (Fig. 4A, top) known for its anti-inflammatory and antiviral properties in viral infections, including SARS-CoV-2 (60–62). An early study showed that glycyrrhizin, of approximately 70% purity, inhibits viral replication and prevents ORF3a-induced HMGB1 release (63). In our study, we explored a range of glycyrrhizin from crude natural extracts to highly purified compounds and tested their effects on ORF3a and conducted a medicinal analysis. The chemical structure of glycyrrhizin consists of glycyrrhetinic acid (enoxolone) (Fig. 4A, left bottom) and a dimer of glucuronic acid (Fig. 4A, right bottom). To determine which components or functional groups are essential for glycyrrhizin activity, we tested 25 different glycyrrhizin compounds and glycyrrhizin structural derivatives (Fig. 4A; Table S1) against ORF3a-induced cell death via MTT assay. Six glycyrrhizin-related compounds (NSC2800, NSC35348, NSC163964, NSC167409, NSC234419, and CAS53956-04-0) inhibited ORF3a-induced cell death in a dose-dependent manner. Among them, NSC234419 (hereafter designated as GL4419) showed the most stable effect with minimal cytotoxicity, effectively suppressing ORF3a-induced apoptosis and necrosis and exhibiting a selectivity index of 22.11 (Fig. 4B and C). Consequently, GL4419 was selected for subsequent studies.

Next, we tested whether GL4419 blocks viral replication. Using a stable baby hamster kidney epithelial BHK21 cell-based SARS-CoV-2 replicon system, which harbors autonomously replicating SARS-CoV-2 RNAs, including ORF3a, and is used for antiviral drug screening (64), we observed a dose-dependent inhibition of viral replication by GL4419 with an EC_50_ of 50.06 ± 5.22 µM (Fig. 4D-a). This inhibitory effect was confirmed in VeroE6 cells using the same glycyrrhizin dose range. Additionally, the WT USA-WA1/2020 virus was used to infect the cells with a titer of 100 TCID_50_ per well of a 96-well plate, and the infected cells were collected at 48 hpi. GL4419 inhibited viral replication, exhibiting an EC_50_ of 271.8 ± 54.57 µM (Fig. 4D-b).

To test whether GL4419 could reverse ORF3a-induced renal cell injury, we treated ORF3a-expressing 293T cells with GL4419 and measured the level of KIM-1 protein production by Western blot analysis. As controls, significant increases in KIM-1 protein levels were observed in both WT and T223I mutant ORF3a-producing cells treated with DMSO. In contrast, GL4419 treatment significantly reduced KIM-1 protein levels (Fig. 4E-a and b). Noticeably, protein levels of ORF3a were also reduced in GL4419-treated cells compared with the DMSO control cells (Fig. 4E-a, first row). To examine whether GL4419 also affects *ORF3a* transcription, RT-qPCR was used to measure the mRNA levels of ORF3a. No significant difference in the levels of ORF3a mRNA transcripts was seen between the DMSO control and GL4419-treated cells (Fig. 4E-c), suggesting that GL4419 reduces ORF3a protein production without affecting its transcription.

Because glycyrrhizin is a known inhibitor of HMGB1 (53), we tested whether ORF3a promotes the nuclear exit of HMGB1, a hallmark of HMGB1 activation (54). As shown (Fig. 4F-a, 1st and 3rd rows), which are mock controls with only DMSO added, HMGB1 remained in the nucleus in the absence of ORF3a in HK2 cells, whereas it completely disappeared from the nucleus in ORF3a-expressing cells, confirming ORF3a-induced HMGB1 activation. However, in ORF3a-producing cells treated with GL4419, HMGB1 proteins remained within the nuclei (Fig. 4F-a, 2nd and 4th rows). Next, we examined whether GL4419 affects nuclear entry of NF-kB, a hallmark of NF-kB activation (58). Consistent with our early experimental result (Fig. 2B), expression of ORF3a in HK2 cells promoted nuclear entry of NF-kB, whereas NF-kB stayed in the cytoplasm when there was no ORF3a present in cells (Fig. 4F-b, 1st and 3rd rows). In contrast, in GL4419-treated cells, NF-kB proteins were retained in the cytoplasm regardless of ORF3a presence (Fig. 4F-b, 2nd and 4th rows). These data suggest that GL4419 not only blocks ORF3a-induced HMGB1 activation but also inhibits subsequent NF-kB activation.

### ORF3a interacts with HMGB1 and alteration of HMGB1 expression influences cellular KIM-1 response and cytokine production

**Fig 5:**
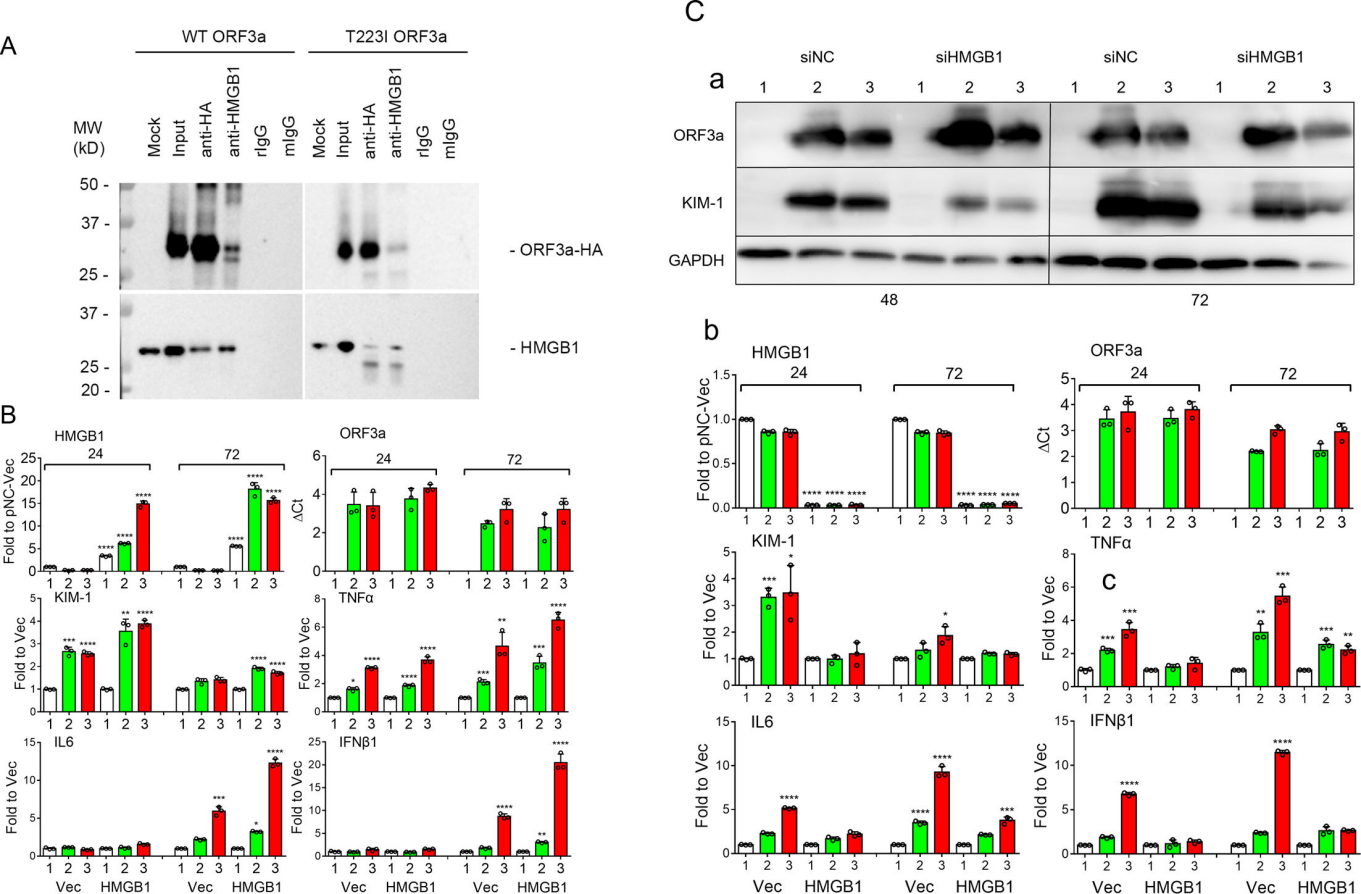
ORF3a interacts with HMGB1 and modulates renal cellular KIM-1 response and cytokine production. (**A)** Interaction of ORF3a with HMGB1 demonstrated by reciprocal co-IP. 293T cells were transfected with WT or T223I mutant ORF3a-carrying plasmid DNA. At 24 hpt, cells were lysed for co-IP analyses. Anti-HA pulldown confirmed the presence of ORF3a and HMGB1, verified by anti-HA and anti-HMGB1 antibodies. Reciprocal IP confirmed ORF3a and HMGB1 interaction. Rabbit IgG (rIgG) and mouse IgG (mIgG) served as negative controls. (**B)** Overproduction of HMGB1 enhances KIM-1 response and cytokine production. 293T cells were co-transfected with HMGB1 and WT or T223I ORF3a plasmids. mRNA levels of HMGB1, ORF3a, *HAVCR1* (KIM-1), and cytokines (TNFα, IL-6, and IFNβ1) were quantified by RT-qPCR at indicated times. Significance: *, *P*  <  0.05; **, *P*  <  0.01; ***, *P*  <  0.001. (**C)** Downregulation of HMGB1 by siRNA reduces KIM-1 response and cytokine production. At 24 hpt, 293T cells were transfected with siRNA targeting HMGB1 or control siRNA, followed by transfection with WT or T223I ORF3a plasmids. Cells were harvested at 24 and 72 hpt. Western blot and RT-qPCR analyses showed successful HMGB1 downregulation, reduced KIM-1, and cytokine levels, more pronounced at 72 hpt.

To determine whether WT and T223I ORF3a proteins interact with HMGB1, we performed reciprocal co-immunoprecipitation (co-IP) experiments in 293T cells. We first pulled down HA-tagged ORF3a proteins using anti-HA antibodies and analyzed them via SDS-PAGE (Fig. 5A). A strong protein band was detected by the anti-HA antibody at the expected size of approximately 32 kDa for HA-tagged ORF3a protein. Controls showed no signals in mock, rabbit IgG (rIgG), and mouse IgG (mIgG) pulldowns, indicating successful and specific HA-ORF3a pulldown. Subsequent immunoblotting with the anti-HMGB1 antibody revealed a distinct protein band at around 29 kDa, an expected protein size for HMGB1. Conversely, HA-ORF3a was also detected in the anti-HMGB1 pulldown protein products, confirming the interaction of WT ORF3a with HMGB1. A similar co-IP profile for the T223I mutant ORF3a and HMGB1 was also observed, suggesting that both WT and T223I mutant ORF3a proteins were associate with HMGB1 in 293T cells.

To elucidate the role of HMGB1 in ORF3a-mediated KIM-1 response and subsequent cytokine production, we conducted two complementary experiments. First, we overproduced HMGB1 in the presence of WT or T223I ORF3a protein by co-transfection. Transfected cells were collected, and the responsive profiles of KIM-1 and cytokines were measured over time as indicated (Fig. 5B). Increased transcription of *hmgb1* was confirmed by RT-qPCR, which did not affect the expression of *ORF3a* (Fig. 5B, 1st row). However, overproduction of HMGB1 triggered an additional and significant increase of KIM-1 at 24 hpt, which waned at 72 hpt (Fig. 5B, 2nd row, left). Consistent with the notion that cytokine production follows KIM-1 elevation, little change in TNFα and no changes in IL-6 and IFNβ1 were seen at 24 hpt. However, a significant increase of these cytokines was observed at 72 hpt. Compared with cells without HMGB1 overproduction, cells with HMGB1 overproduction displayed an additional and significant increase in cytokine production beyond control cells (Fig. 5B, 2nd row, right, and 3rd row, left).

In contrast to HMGB1 overproduction, we also tested the effect of *hmgb1* downregulation on ORF3a-mediated KIM-1 response and the respective cytokine production under the same experimental timeframe. The successful downregulation of *hmgb1* transcription and protein production by small interference RNA (siRNA) was verified by both Western blot (Fig. 5C) and RT-qPCR analyses (Fig. 5C, first row). Although downregulation of *hmgb1* had no clear effect on ORF3a expression (Fig. 5C, 1st row, right), it significantly reduced the level of KIM-1 protein back to near background levels (Fig. 5C, 2nd row, left). Consistently, the levels of respective cytokine production were also significantly reduced (Fig. 5C, 2nd row, right, and 3rd row, left).

Together, these data suggest that ORF3a triggers the cellular KIM-1 response and subsequent cytokine production primarily through its interaction with HMGB1 during the induction of renal cell injury.

## DISCUSSION

In this study, we elucidated a functional link between SARS-CoV-2 infection, viral protein ORF3a expression, and CAKI. Specifically, we discovered a correlation between the presence of ORF3a and inflammation-associated apoptotic death of renal cells in the kidneys of COVID-19 individuals with CAKI (Fig. 1). Furthermore, using renal cell lines, such as 293T and HK2 *in vitro*, we demonstrated that ORF3a not only independently induces renal cell cytotoxicity (Fig. 2) but also triggers a kidney cell injury-specific response. This response was validated by the elevation of the kidney injury-specific biomarker KIM-1 (Fig. 2B) (22, 23), and apoptotic cell death (Fig. 2D), facilitated by the activation of NF-kB-mediated proinflammatory cytokine production (TNFα, IL-6, and IFNβ1) (Fig. 2C). Note that although 293T cells are not professional immune cells, they can produce both pro-inflammatory and anti-inflammatory cytokines (27, 28, 65, 66). Notably, although IFNβ1 is typically considered as an anti-inflammatory type I cytokine, its expression levels coincide with pro-inflammatory TNFα and IL-6 (Fig. 2C-b). Because IFNβ1 can also exhibit pro-inflammatory properties under certain conditions (67, 68), it is currently unclear whether IFNβ1 acts as a pro- or anti-inflammatory agent despite its elevation upon ORF3a production. To resolve this question, in the future, we could test the effect of IFNβ1 by either overexpressing or downregulating it in the context of ORF3a-induced apoptotic cell death.

Both our study and previous research have demonstrated that ORF3a expression induces renal cell apoptosis and necrosis through NF-kB-mediated cytokine production (27, 28, 32). Therefore, finding an association between ORF3a expression and inflammation-associated renal cell death in the kidneys of COVID-19 individuals with CAKI (Fig. 1) is not unexpected. What is unique is the agreement between the clinical findings described here and our *in vitro* results, establishing a robust correlation between SARS-CoV-2 infection, ORF3a expression, and CAKI. In addition, we showed that ORF3a-induced renal cell death caused typical kidney injury as KIM-1 was specifically elevated upon ORF3a expression (Fig. 2B). Moreover, the elevation of KIM-1 may be a general renal cellular response to all ORF3a proteins as both the WT and Omicron variant-associated T223I mutant ORF3a proteins trigger the elevation of KIM-1 in a similar manner (Fig. 2B), and these two ORF3a proteins represent the two distinctive ORF3a protein types we have identified thus far (Fig. 2A) (28, 33).

From a mechanistic perspective, it was postulated that ORF3a induces renal cell-specific injury partly through IL-6 and TRIM59 (Tripartite motif 59)-mediated STAT3 activation (32). Interestingly, prior interactome analyses have predicted an interaction between ORF3a and TRIM59 (69, 70). Indeed, we validated this interaction and further demonstrated that TRIM59, a primary ER-associated ubiquitin E3 ligase, is associated with 26S proteasome-mediated protein degradation of ORF3a (28). Thus, it is plausible that, in addition to activating STAT3, TRIM59 may also serve as a cellular host response to counteract the effects of ORF3a (70, 71).

To examine whether a similar correlation exists between ORF3a and kidney injury in SARS-CoV-2-infected mice, we employed a K18-hACE2 transgenic mouse model (51). Our observations in these mice revealed an association similar to what we observed in humans (Fig. 1), linking ORF3a-induced cytotoxicity with kidney injury (Fig. 3A). Additionally, we confirmed ORF3a-induced kidney-specific injury by replicating the effect through direct adenoviral injection into the mouse kidneys (Fig. 3B). Overall, our animal study results align with our immunochemical findings in the kidney tissues of COVID-19 individuals with CAKI (Fig. 1), further substantiating the role of ORF3a in contributing to renal cell and tissue damage as well as to CAKI (Fig. 3A).

Early mouse model studies indicated that SARS-CoV-2 infection results in kidney damage, characterized by tubular damage and focal tubular collapse, mirroring observations in COVID-19 patients (32, 45, 72, 73). Additionally, the deletion of *ORF3a* from the viral genome has been associated with reduced tissue damage, such as in the lungs, suggesting a potential role of ORF3a in tissue damage. Notably, however, kidney tissues were not examined in those animal studies.

Apart from the connection between ORF3a protein and CAKI, the SARS-CoV-2 nucleocapsid (N) protein also induces renal tubular epithelial cell death, leading to AKI (72). Interestingly, the N protein interacts with Smad3 and promotes TGF-β/Smad3 signaling, leading to renal tubular epithelial cell death and AKI through cell cycle G1 arrest. N protein-induced AKI is exacerbated in diabetic kidneys of db/db mice (74). Treatment of N-induced AKI in db/db mice with the natural compound quercetin reverses Smad3-mediated cell cycle G1 arrest, thereby improving AKI (74). Although the relationship between ORF3a-induced CAKI and that induced by N protein remains unclear, it is noteworthy that quercetin is also an HMGB1 inhibitor (75, 76). Given that quercetin and glycyrrhizin have different modes of action, it would be interesting to test whether quercetin can be used alongside glycyrrhizin to further mitigate ORF3a-induced CAKI. Nevertheless, these reports suggest that the results of our animal studies may not fully reflect the potential impact of ORF3a on kidney injury. Additionally, the mice we utilized were normal mice without pre-existing kidney disease, and most of the SARS-CoV-2-infected K18-hACE2 Tg mice succumbed to infection approximately 1 week post-infection in our experiments and in related studies (51). Therefore, the kidney tissues we examined from the surviving mice may underestimate the impact of ORF3a on kidney injury. In a study involving an animal coronavirus MHV-1 mouse model, a comparison of the acute and long-term impacts of viral infection showed severe kidney involvement during acute viral infection, leading to pronounced kidney fibrosis after prolonged viral infection (77). Hence, to comprehensively understand the effects of ORF3a on CAKI, future investigations should consider testing infected K18-hACE2 Tg mice over an extended period of time.

Glycyrrhizin is a well-known natural compound recognized for its anti-inflammatory and antiviral properties against various viral infections, including SARS-CoV-2 (60–62). In our search for ORF3a inhibitors, the glycyrrhizin analogs were selected among others from the National Cancer Institute (NCI) chemical database (A portion of the NCI database is available to the public at https://cactus.nci.nih.gov/ncidb2.2/). We confirmed that both essential components of glycyrrhizin, the enoxolone and dimer of glucuronic acid, are required for its inhibitory activity against ORF3a (Fig. 4A). Additionally, none of the analogs tested has shown better activity than glycyrrhizin, NSC-234419 (GL4419) CAS Registry No.1405–86-3. We further showed that GL4419 is the most stable and could potentially serve as a therapeutically viable ORF3a inhibitor with a selective index of 22.11 (Fig. 4B). GL4419 effectively suppresses ORF3a-induced apoptosis and necrosis (Fig. 4B and C) and blocks viral replication in hamster and monkey kidney epithelial cells (Fig. 4D). An earlier study utilizing a relatively low purity of glycyrrhizin reported similar findings (63). Beyond these initial findings, we further demonstrate that GL4419 alleviates ORF3a-induced renal cell injury, as evidenced by the reduction in KIM-1 protein levels in GL4419-treated, ORF3a-producing cells (Fig. 4E). Mechanistically, GL4419 may inhibit ORF3a at the translational level through direct protein degradation, as a significant decrease in ORF3a protein levels was also observed along with the reduction of KIM-1 protein levels at 24 hpt (Fig. 4E-a and b). However, the ORF3a mRNA remained at similar levels with or without GL4419 treatment even at 48 hpt (Fig. 4E-c), suggesting that GL4419 does not affect the transcription of ORF3a. The exact mechanism by which GL4419 mediates ORF3a protein degradation remains unknown. Our early study showed that ORF3a protein is subject to ubiquitin E3 ligase TRIM59 and 26S proteasome-mediated protein degradation (28). Furthermore, ORF3a proteins primarily associated with the ER (E-ORF3a) display more robust protein degradation than those associated with lysosomes (L-ORF3a) (28). Interestingly, there was no clear protein degradation of either WT or T223I ORF3a in the early hours up to 24 hpt without GL4419 treatment (Fig. 2B; Fig. 4E-a). Conversely and consistent with our earlier findings, the ER-associated T223I mutant protein showed a stronger reduction in ORF3a protein levels than the lysosome-associated WT protein in GL4419-treated cells (Fig. 4E-a and b ). This result suggests that GL4419 may promote ORF3a protein degradation through the same 26S proteasome-mediated mechanism. Additionally, GL4419 may inhibit ORF3a through direct interaction as early NMR and fluorescence analyses suggested such a possibility (78). Notably, the inhibitory activity of GL4419 is likely a general effect against ORF3a proteins residing in either lysosomes or the ER, as it inhibits both the WT ORF3a and the T223I mutant, representing two distinctive ORF3a protein types detected so far (Fig. 2A) (28, 33).

Glycyrrhizin is a known inhibitor of HMGB1 (53). As HMGB1 acts as a DAMP molecule in response to cellular stress or tissue damage (54), it suggests that the function of HMGB1 may be related to or possibly interact with ORF3a. Indeed, ORF3a physically interacts with HMGB1 (Fig. 5A). Moreover, upon ORF3a production, HMGB1 was released from the nucleus into the cytoplasm (Fig. 4F-a), which was associated with nuclear entry of the RelA/p65 subunit of NF-kB, an indicator of NF-kB activation (Fig. 4F-b). Significantly, treatment of ORF3a-producing cells with GL4419 blocked nuclear exit of HMGB1 and nuclear entry of p65, suggesting that the inhibitory effect of GL4419 on ORF3a was likely achieved through the inhibition of HMGB1 activation and NF-kB-mediated cytokine production (Fig. 4F). Because ORF3a interacts with HMGB1 (Fig. 5A), and GL4419 also binds to HMGB1 (78), in the future, it would be interesting to test whether the interaction of ORF3a with HMGB1 can be blocked by GL4419.

Our data further suggest that HMGB1 plays a critical role in ORF3a-inflicted kidney injuries, as overproduction or downregulation of HMGB1 protein production resulted in corresponding changes in renal cellular KIM-1 response and cytokine production (Fig. 5B and C). These findings are consistent with earlier reports showing that HMGB1 plays a crucial pathological role in inflammation-mediated kidney injury and diseases (55) and that therapeutic inhibition of HMGB1 by glycyrrhizin improves long-term recovery of patients with AKI (56).

Besides inhibiting ORF3a, glycyrrhizin also inhibits SARS-CoV-2 replication by targeting the S1 protein (63). Although no report has yet demonstrated a direct interaction between SARS-CoV-2 ORF3a and the S protein, ORF3a has been shown to interact with the S protein of SARS-CoV (79, 80). Hence, it would be intriguing to investigate whether the SARS-CoV-2 ORF3a protein binds to the S protein and whether glycyrrhizin blocks viral replication by affecting the interaction of ORF3a with the S protein.

In conclusion, we have uncovered a direct functional link between SARS-CoV-2 viral ORF3a protein and COVID-19-associated kidney injury, underscoring ORF3a as a unique therapeutic target for alleviating CAKI using ORF3a inhibitors such as glycyrrhizin.

## MATERIALS AND METHODS

### Viral infection and injection of animals

A total of 12 K18-hACE2 Tg mice [strain B6.Cg-Tg(K18-ACE2)2Prlmn/J, strain #: 034860, The Jackson Laboratory] were intranasally infected with SARS-CoV-2 New York strain at the dose of 2.5 × 10^4^ TCID_50_ (51). Two surviving mice were euthanized on day 8 p.i*.*. Kidney tissues were fixed with formalin and processed for paraffin embedding and sectioning to evaluate ORF3a protein level and kidney tissue damage using IHC compared with controls. The K18-hACE2 mice were housed under pathogen-free conditions, and viral infection was conducted in a biosafety level 3 (BSL-3) facility at the University of Maryland College Park.

To investigate the correlation between *in vitro* cytopathic effects of ORF3a and CAKI *in vivo*, we delivered ORF3a into C57BL/6 mouse kidneys via injection of an adenoviral vector carrying ORF3a (Ad5-ORF3a). The procedure followed a previously described protocol (32), where mice were initially anesthetized, and the left renal pedicle was occluded. Subsequently, 100 µL of concentrated Ad5-ORF3a (viral titer approximately 3.0 × 10^6^ TU/μL) was injected into the lower pole of the left kidney, progressing to the upper pole along the kidney’s long axis. The occlusion of the renal pedicle was then reversed. Control mice received Ad5-DsRed injected into the same kidney region. C57BL/6 mice were housed under pathogen-free conditions at the animal facility, University of Maryland School of Medicine, Baltimore.

### Immunostaining of kidney tissues

Immunostaining of human biopsy kidney tissues has been previously described (19). For mouse kidney tissues, the procedure followed established protocols (32, 81). Briefly, kidneys from C57BL/6 or K18-hACE2 transgenic mice and WT controls were fixed in formalin and embedded in paraffin. Sections, 10 µM thick, were cut from the paraffin-embedded blocks containing the entire kidney by using a microtome. These sections were deparaffinized and rehydrated through graded ethanol (100%, 95%, and 70%) and xylene washes. Following antigen retrieval, the sections were blocked in 5% donkey serum in phosphate-buffered saline (PBS) with 0.2% Triton-X. For all immunolabelings, the sections were incubated at 4°C overnight with primary antibodies directed against anti-cleaved caspase 3 (Cell Signaling: 9661S), anti-KIM-1 (Invitrogen: PA5-98302), anti-ORF3a (LS Bio: LS-C829863), anti-TNFα (Santa Cruz Biotechnology: sc-1350), or anti-NF-kB (p65) (Santa Cruz Biotechnology: sc-372). After several rinses in PBS, the sections were incubated with species-appropriate fluorescent secondary antibodies (Alexa Fluor 488 and 555, Molecular Probes, Thermo Fisher Scientific, Waltham, MA, USA) for 1 h at room temperature. Controls for IHC included the omission of primary antibodies. Unbiased assessments of specific labeling were obtained using NIS-Elements AR software (Nikon Instruments, Melville, NY, USA) from sections (one section per mouse) immunolabeled as a single batch. All images for a given signal were captured using uniform parameters of magnification, area, exposure, and gain.

### Cell lines, growth media, and plasmid transfection

Two human renal cell lines were utilized in this study: the human embryonic epithelial cell line HEK293T (ATCC CRL-1573) and the human renal proximal tubule epithelial cell line HK2 (ATCC CRL-2190). HEK293T (a.k.a. 293T) cell line was maintained in Dulbecco’s Modified Eagle’s Medium (DMEM; Corning Cat#: 10-017-CV), supplemented with 10% fetal calf serum (FBS, Gibco: Cat#: 100-438-026), penicillin (100 IU/mL), and streptomycin (100 µg/mL). The HK2 cell line, however, was cultured in keratinocyte serum-free medium (K-SFM; Life Technologies Cat#: 17005-042), which enhances adherence to culture dishes. For immunofluorescence assays, a 1:1 mixture of K-SFM and DMEM was used for HK2 cells, as it improves adherence (28). A baby hamster kidney BHK21 cell line harboring SARS-CoV-2-replicon containing a NanoLuc-Neo reporter and NSP1 mutations (K164A/H165A) was obtained through BEI Resources (Catalog#: NR-58876). BHK21 cells were maintained in DMEM supplemented with 10% FBS and 200 µg/mL G418. All cell lines were maintained in a 37°C incubator with 5% CO_2_. Plasmids of interest were transfected into 293T or HK2 cells using Lipofectamine 3000 according to the manufacturer’s instructions (Thermo Fisher Scientific).

### Plasmid construction and adenovirus packaging

The pCAG plasmid carrying a HA-tag WT ORF3a was described previously (33). The T223I mutant was introduced into WT ORF3a using the overlapping PCR method and the primers listed in Table S2. The amplified fragment was inserted into the pCAG backbone via EcoRV/AgeI sites using the Gibson Assembly Cloning Kit (NEB). pcDNA3.1 Flag hHMGB1 was a gift from Yasuhiko Kawakami (Addgene plasmid #31609).

To generate the adenoviral vectors, pAdenoX-DsRed-ORF3a and pAdenoX-DsRed-Vec were generated using the In-Fusion Cloning Kit (Takara). ORF3a or 47-bp annealing oligos with more than 15-nt overlap sequence with the backbone were inserted into the linearized AdenoX-DsRedExpress plasmid (Takara). Once the final constructs were confirmed by restriction enzyme digestion and Sanger sequencing, the plasmids were linearized by the *Pac*I restriction enzyme and transfected into AD293 cells to produce the recombinant adenovirus Ad5-DsRed-ORF3a and Ad5-DsRed-Vec following the Adeno-X user manual (Takara). The primers or oligos are listed in Table S2.

### Viruses and viral infection

In this study, the SARS-CoV-2 reference strain USA-WA1/2020 (Genbank accession number: MN985325) and strain New York-PV08410/2020 (GenBank accession number: MT370900) were used. For viral infection, cells were seeded on coverslips in 12- or 24-well cell culture plates and allowed to grow overnight until reaching 90% confluency. Subsequently, the cell culture plates were transferred to an ABSL-3 facility for virus inoculation. The virus was diluted to the desired multiplicity of infection (MOI) and added to the cells, followed by incubation for the specified duration before fixation for further analysis. Additionally, a stable baby hamster kidney BHK21 cell-based SARS-CoV-2 NanoLuc replicon system was utilized to assess the impact of glycyrrhizin on viral replication. This system contains autonomously replicating SARS-CoV-2 RNAs, including ORF3a and employs a Nano-Glo Luciferase Assay (Promega), following established protocols (64).

### Immunofluorescent assay and confocal microscopy

The procedure for immunofluorescent assay has been previously detailed (33). Briefly, cells were cultured on coverslips and fixed with 1% paraformaldehyde for 10 min at room temperature. Following fixation, cells were permeabilized with 0.2% Triton-X100 for 20 min on ice. Subsequently, a series of sequential incubations were conducted. Cells were first exposed to primary antibodies, followed by incubation with Texas Red (TR)- or FITC-labeled secondary antibodies, all diluted in PBS for 30 min each. After incubations, cells were rinsed in PBS, stained with Hoechst 33258 (0.5 μg/mL) to visualize DNA, and mounted using Fluoromount-G (Fisher Scientific, Newark, DE) for microscopic analysis.

### Co-immunoprecipitation, immunoblot analysis and antibodies

Co-IP and immunoblot analyses were conducted following previously established protocols (28, 33). Briefly, WT or T223I mutant *ORF3a*-expressing plasmids were transfected into 293T cells. At the specified time post-transfection, cells were lysed with ice-cold RIPA lysis buffer (Millipore Sigma) and kept on ice for 10 min. After centrifugation at 3,000*g* for 5 min, the supernatants were transferred to new tubes.

For co-IP analysis, whole cell lysates were incubated with anti-HA antibodies for ORF3a-HA pulldown or anti-HMGB1 antibodies for HMGB1 pulldown. Rabbit IgG (rIgG) and mouse IgG (mIgG) served as negative controls for rabbit and mouse antibodies, respectively. These mixtures were then combined with protein G-Sepharose beads (Amersham Pharmacia Biotech AB, Sweden) and incubated for 3 h according to the manufacturer’s instructions. Following immunoprecipitation, beads were washed thrice with PBS containing 0.1% bovine serum albumin and a protease inhibitor cocktail. Immunoprecipitated complexes were eluted in PBS mixed with 2× Laemmli buffer (20 µL each), heated briefly at 95°C for 5 min, and centrifuged to separate beads, which were then subjected to SDS-PAGE and subsequent immunoblotting analysis.

For immunoblotting, equal amounts of total protein were separated on SDS-PAGE gels by electrophoresis and transferred onto polyvinylidene difluoride (PVDF) membranes (Bio-Rad). The following antibodies were used for protein detection: mouse anti-ORF3a (R&D Systems: MAB10706), anti-KIM-1 (Invitrogen: YH4016831), and mouse anti-GAPDH (Cell Signaling: Cat# 2118).

### Measurement of cell growth, viability, and apoptotic cell death

To measure cell growth, viability, and apoptotic cell death, 293T cells were initially seeded at a density of 2.4 × 10^4^ cells per well in a 96-well plate. Cells were allowed to adhere and adapt overnight at 37°C in a 5% CO_2_ atmosphere. Subsequently, 100 ng of plasmid DNA was introduced into the cells using Lipofectamine 3000 following the manufacturer’s protocol. At 48 hpt, cell growth and death were quantified by cell counting and trypan blue staining. Cells were trypsinized, mixed with an equal volume of trypan blue staining solution, and counted using a TC20 automated cell counter (Bio-Rad) to determine the total cell count and the number of non-viable (stained) cells. Cell viability was assessed using the MTT assay. Briefly, 10 µL of a 5 mg/mL MTT solution was added to each well and incubated at 37°C for 2 to 5 h. After removing the medium, 100 µL of DMSO was added to solubilize the formazan crystals, and the plates were further incubated at 37°C for 15 min. The absorbance at 570 nm was measured immediately using an H1M microplate reader (Agilent). Cell apoptosis and necrosis were measured using a RealTime-Glo annexin V apoptosis and necrosis assay kit (Promega) (27).

### Measurement of NF-κB promoter activity

To assess NF-κB promoter-mediated transcriptional activity, an NF-κB luciferase assay was conducted following a previously published protocol (27). Initially, 2.4 × 10^4^ 293T cells were seeded into individual wells of a white 96-well plate and incubated overnight. For the assay, co-transfection included 0.05 µg of the plasmid of interest, 0.05 µg of pNF-κB-Luc, and 0.01 µg of pRL-SV40. To quantify luciferase activity, a dual luciferase reporter assay system (Promega: Cat# E1910) was utilized with measurements taken using the Synergy H1 microplate reader from Agilent at specified time intervals. Signal normalization was achieved using Renilla luciferase as an internal control. Fold changes were calculated relative to the control containing an empty vector.

### Reverse transcription-quantitative PCR (RT-qPCR)

RT-qPCR was performed following a previously described methodology (27). Initially, 293T cells were cultured in a six-well plate until reaching approximately 70% confluency. Cells were transfected with 2.5 µg of plasmid DNA. At 24, 48, and 72 hpt, cells were harvested, and RNA was extracted using Trizol (Invitrogen, 448706). The extracted RNA was treated with RQ1 RNase-Free DNase (Promega: M6101) to eliminate genomic DNA contamination. Subsequently, the RNA was reverse transcribed into cDNA using reverse transcriptase (Thermo Fisher: 4311235). Real-time PCR was conducted on a QuantStudio 3 Real-time PCR system using gene-specific primers and SYBR Green Master Mix (Thermo Fisher: A46109) to detect mRNA expression levels. The amplification conditions included 40 cycles of 95°C for 10 s and 60°C for 30 s, followed by a melting curve analysis. Fold-change in mRNA expression was calculated using the 2^-ΔΔCT^ method, with GAPDH mRNA serving as the internal control.

### siRNA treatment

293T cells were prepared as previously described. At time 0, 25 pM of *hmgb1*-specific siRNA (HMGB1: SASI_Hs01_0019–6036/HMGB, Sigma) or a Mission siRNA Universal Negative Control #1 (siNC, Sigma: SIC001) was added. At 24 h post-siRNA addition, the cells were transfected with either a WT or T223I mutant *ORF3a*-carrying pCAG plasmid, or co-transfected with an *ORF3a*-carrying plasmid and an *hmgb1*-carrying plasmid. Transfected cells were harvested at 24, 48, and 72 hpt. Cellular proteins were extracted as described previously and subjected to Western blot analysis. Concurrently, total cellular RNA was isolated and used for quantitative RT-PCR analysis, following methods outlined above and in reference 27.

### Medicinal analysis of glycyrrhizin and drug treatment

All glycyrrhizin-related compounds were provided by the Developmental Therapeutics Program of the National Cancer Institute, the National Institutes of Health or purchased directly from commercial sources (Table S1). The powdered compounds were dissolved in DMSO and stored at −20°C as a 50 mM stock solution. GL4419 (NSC234419), with a molecular weight of 822.93 and molecular formula of C42H62O16, was utilized in all subsequent drug treatment experiments.

### Statistical analysis

Statistical analyses were conducted using Prism 9 software (GraphPad, San Diego, CA, USA). Pairwise *t*-tests, one-way ANOVA, or two-way ANOVA were employed as appropriate. Statistical significance was defined as *P* < 0.05 at the 95% confidence level. Symbols *, **, ***, and **** were used to denote significance levels: *P* < 0.05, *P* < 0.01, *P* < 0.001, and *P* < 0.0001, respectively.

## SUPPLEMENTAL MATERIAL

Table S1 mbio.02308-24-s0001.pdf Glycyrrhizin-related compounds tested in this study.

Table S2 mbio.02308-24-s0002.pdf Oligonucleotide primers used in this study.

ASM does not own the copyrights to Supplemental Material that may be linked to, or accessed through, an article. The authors have granted ASM a non-exclusive, world-wide license to publish the Supplemental Material files. Please contact the corresponding author directly for reuse.
