# Supplementary material for: SARS-CoV-2 ORF3a induces COVID-19-associated kidney injury through HMGB1-mediated cytokine production
Source: mBio. 2024 Sep 30;15(11):e02308-24. doi: 10.1128/mbio.02308-24 (PMC11559048; doi:10.1128/mbio.02308-24)
Supplement: Table S1 — Glycyrrhizin-related compounds tested in this study. [file mbio.02308-24-s0001.pdf]

Table S1. Glycyrrhizin related compounds tested in this study.

Compound Number	Compound Structure	NSC Number	Molecular Weight	ORF3a Inhibition (Y/N)
1	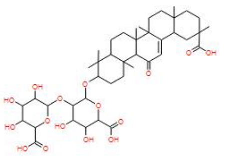	2800	839.96	Y
2	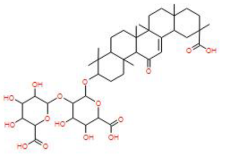	35348	839.96	Y
3	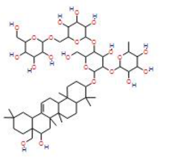	104798	1091.28	N
4	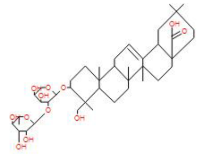	106553	750.96	N
5	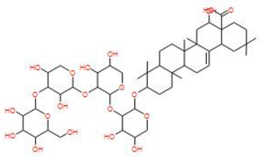	106554	1031.18	N
6	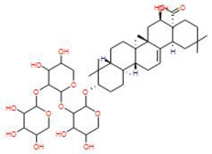	106555	869.04	N
7	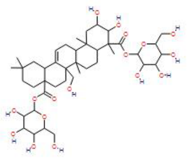	124670	842.96	N

8	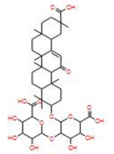	163964	845.92	Y
9	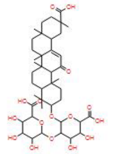	167409	822.93	Y
10*	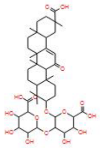	234419	822.93	Y
11	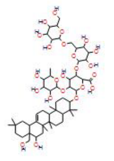	409073	1105.26	N
12	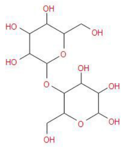	2559	342.30	N
13	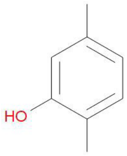	2599	122.16	N
14	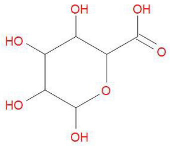	9248	194.14	N
15	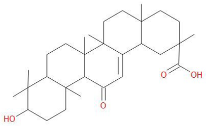	35347	470.68	N

16	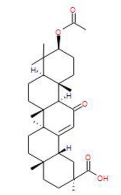	35349	512.72	N
17	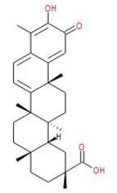	70931	450.61	N
18	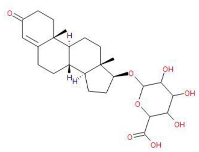	92192	464.55	N
19	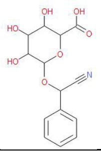	103055	309.27	N
20	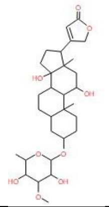	123977	550.68	N
21	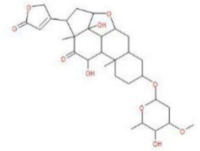	123978	562.65	N
22	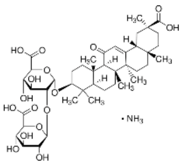	CAS#: 53956-04-0	839.96	Y
23	SNA	NA	NA	N
24	SNA	NA	NA	N
25	SNA	NA	NA	N

Note: *, This ORF3a inhibitor was selected for further studies and designated as GL4419. SNA, structure not available; NA, not available. Compounds 1-21 were provided by the Developmental Therapeutic Program, NCI/NIH. Compound 22 was purchased from Sigma (G2137), and compounds 23-25 were purchased as natural licorice extracts from Earthborn Elements, TrueGether and FabLab, respectively.
