# Supplementary material for: SARS-CoV-2 ORF3a induces COVID-19-associated kidney injury through HMGB1-mediated cytokine production
Source: mBio. 2024 Sep 30;15(11):e02308-24. doi: 10.1128/mbio.02308-24 (PMC11559048; doi:10.1128/mbio.02308-24)
Supplement: Table S2 — Oligonucleotide primers used in this study. [file mbio.02308-24-s0002.pdf]

Table S2. Primers used in this study.

pCAG-ORF3a-F	CAGTGTGGTGGAATTCTGCAGATATCGCCACCATGGATTTGTTTATGAGAATC
ORF3a-LE-HA-R	GTAGTCAGGCACGTCGTAAGGATACTCGAGCAAAGGCACGCTAGTAGTCG
HA-pCAG-R	CAATGGTGATGGTGATGATGACCGGTTTAGGCGTAGTCAGGCACGTCGTAAG GATA
T223I-OL-F	CAACTCAATTGAGTACAGACAtTGGTGTTGAACATGTTACCTTC
T223I-OL-R	GAAGGTAACATGTTCAACACCAaTGTCTGTACTCAATTGAGTTG
pADX-EV-F	GCGTAACTATAACGGTCATCGATATTTAAATGGAGAAAGAGGTAATG
pADX-EV-R	CATTACCTCTTTCTCCATTTAAATATCGATGACCGTTATAGTTACGC
pADX-ORF3a-F	GACGCGTAACTATAACGGTCGCCACCATGGATTTGTTTATGAGAATC
pADX-ORF3a-R	ATTTCATTACCTCTTTCTCCTTACAAAGGCACGCTAGTAGTCG
KIM-1-qF	CCAGCAGAAACCCACCCTAC
KIM-1-qR	GGTGTCATTCCCATCTGTTGT
NF-kB1-qF	CACCCTGACCTTGCCTATTT
NF-kB1-qR	AGGTCCATCTCCTTGGTCT
HMGB1-qF	TGCAAACTTGTCGGGAGGAG
HMGB1-qR	TAGCAGACATGGTCTTCCACC
TNFα-qF	CGAGTGACAAGCCTGTAGC
TNFα-qR	GGTGTGGGTGAGGAGCACAT
IL-6-qF	AATAACCACCCCTGACCCAAC
IL-6-qR	AATCTGAGGTGCCCATGCTAC
IFNB1-qF	GGCAGTATTCAAGCCTCCCAT
IFNB1-qR	TCTCCTGTTGTGCTTCTCCAC
ORF3a-qF	GATGGCTTATTGTTGGCGTTG
ORF3a-qR	ACAAAGTGAACACCCTTGGAGAG
GAPDH-qF	GTCAGTGGTGGACCTGACCT
GAPDH-qR	TGAGCTTGACAAAGTGGTCG
